# Rac1 regulates pancreatic islet morphogenesis

**DOI:** 10.1186/1471-213X-9-2

**Published:** 2009-01-06

**Authors:** Thomas U Greiner, Gokul Kesavan, Anders Ståhlberg, Henrik Semb

**Affiliations:** 1 Stem Cell and Pancreas Developmental Biology, Stem Cell Center, Lund University, BMC B10, Klinikgatan 26, SE-221 84 Lund, Sweden; 2 Department of Clinical Neuroscience and Rehabilitation, Institute of Neurosciences and Physiology, Sahlgrenska Academy at Göteborg University, Medicinaregatan 9A, SE 413 90 Göteborg, Sweden; 3 TATAA Biocenter, Odinsgatan 28, 411 03 Göteborg, Sweden

## Abstract

**Background:**

Pancreatic islets of Langerhans originate from endocrine progenitors within the pancreatic ductal epithelium. Concomitant with differentiation of these progenitors into hormone-producing cells such cells delaminate, aggregate and migrate away from the ductal epithelium. The cellular and molecular mechanisms regulating islet cell delamination and cell migration are poorly understood. Extensive biochemical and cell biological studies using cultured cells demonstrated that Rac1, a member of the Rho family of small GTPases, acts as a key regulator of cell migration.

**Results:**

To address the functional role of Rac1 in islet morphogenesis, we generated transgenic mice expressing dominant negative Rac1 under regulation of the Rat Insulin Promoter. Blocking Rac1 function in beta cells inhibited their migration away from the ductal epithelium *in vivo*. Consistently, transgenic islet cell spreading was compromised *in vitro*. We also show that the EGF-receptor ligand betacellulin induced actin remodelling and cell spreading in wild-type islets, but not in transgenic islets. Finally, we demonstrate that cell-cell contact E-cadherin increased as a consequence of blocking Rac1 activity.

**Conclusion:**

Our data support a model where Rac1 signalling controls islet cell migration by modulating E-cadherin-mediated cell-cell adhesion. Furthermore, *in vitro *experiments show that betacellulin stimulated islet cell spreading and actin remodelling is compromised in transgenic islets, suggesting that betacellulin may act as a regulator of Rac1 activity and islet migration *in vivo*. Our results further emphasize Rac1 as a key regulator of cell migration and cell adhesion during tissue and organ morphogenesis.

## Background

All three major pancreatic cell types, including acinar, ductal and endocrine cells, originate from common Insulin promoter factor (Ipf1)/Pancreatic and duodenal homeobox 1 (Pdx1) -expressing progenitors within the posterior foregut endoderm. Initially, these cells evaginate from the foregut endoderm to form the dorsal and ventral pancreatic buds that later fuse to form the proper pancreas. The pancreatic epithelium proliferates and branches into the surrounding mesenchyme to form a highly branched epithelial sheet [1,2]. Concomitant with branching morphogenesis, cells of the pancreatic ductal epithelium differentiate into Neurogenin 3 (Ngn3)-expressing endocrine precursors through regulation of Notch signalling [3,4]. During the secondary transition which starts around embryonic day (E)13, these Ngn3-positive progenitors differentiate into hormone-producing islet cells which delaminate and migrate out into the surrounding mesenchyme to initiate clustering into vascularised islets of Langerhans, consisting of the α-, β-, ε-, δ- and PP-cells [5,6].

Although morphological evidence for delamination of endocrine cells from ducts was first shown by Pictet and Rutter [6], the cellular and molecular mechanisms underlying the delamination and migration processes are poorly understood. Regarding the potential mechanisms for delamination, it has been proposed that it may involve breaking down the basal lamina, e.g. through the activity of matrix metalloproteinases (MMPs) [7]. This hypothesis was tested by analysing mice deficient for MMP2 and MMP9 or overexpressing TIMP1, an inhibitor of MMPs, during pancreas development. However neither endocrine cell delamination nor islet cell migration was effected in these mice [8]. In fact, these results are consistent with electron micrographs [6] which provide morphological evidence for cells not breaking through the basal lamina during delamination, but rather budding off with a piece of the basal lamina intact around the delaminating endocrine cells. Thus, other processes are likely to be responsible for islet cell delamination during islet morphogenesis. Insights into the potential mechanisms for islet cell migration have been contributed by gene ablation studies demonstrating that Wnt5a and the epidermal growth factor (EGF)-receptor are required for migration of newly formed β cells from the ductal epithelium [9,10]. However, the mechanism of action remains unknown. Furthermore, the fact that these mice succumb to other severe phenotypes shortly after birth, suggests that it cannot be excluded that the pancreas phenotype is secondary to other phenotypes.

Cell migration requires dynamic regulation of cell-cell and cell-extracellular matrix (ECM) adhesion, as well as of cytoskeletal rearrangement. Pioneering work highlight Rho-GTPases as key regulators of actin cytoskeleton dynamics and lamellipodia formation [11,12]. The Rho family of small GTPases act as molecular switches, cycling between an active GTP-bound and an inactive GDP-bound state [13]. Numerous reports have demonstrated an active role of the Rho-GTPase member Rac1 in cell migration and cell-cell adhesion in cultured cells [14-16]. Rac1 is ubiquitously expressed *in vivo *[17] and Rac1 null mutant mice show severe developmental defects, including failure to form the three germ layers during gastrulation [18], precluding studies of its role in later processes such as organogenesis. In Drosophila, Rac1 regulates both tubulogenesis and dorsal closure [19-21], suggesting an important *in vivo *role of Rac1 in both cell migration and cell-cell adhesion. Notably, a common denominator when Rac1 function is perturbed is consequences on E-cadherin-mediated cell-cell adhesion [15]. However, inconsistent results, such as Rac1 being capable of both promoting and inhibiting E-cadherin mediated cell-cell adhesion, make it difficult to extract a clear picture of how Rac1 regulates E-cadherin function *in vivo *[22,23].

To shed more light on the *in vivo *function of Rac1 as a regulator of cell-cell adhesion and cell migration during tissue formation and organogenesis, we have addressed its role in pancreatic islet morphogenesis. For this purpose, transgenic mice were generated where a dominant negative form of Rac1 (RacN17) was expressed under regulation of the Rat Insulin Promoter (RIP-RacN17). RacN17 has been extensively used to study Rac1 function both *in vitro *and *in vivo *(reviewed by [14,16]). RacN17 has a low affinity for GTP and thereby remains in its inactive GDP bound state. By binding to upstream activating exchange factors (GEFs) it is believed to prevent these factors from activating endogenous Rac1 [24].

We identify Rac1 as a key signalling molecule regulating migration of endocrine cells away from the ductal epithelium. Changes in the expression and levels of cell-cell contact E-cadherin, suggest that the mechanism of action may involve control of E-cadherin-mediated cell-cell adhesion. Furthermore, *in vitro *experiments show that betacellulin stimulated islet cell spreading and actin remodelling is compromised in transgenic islets, suggesting that betacellulin may act as a regulator of Rac1 activity and islet migration *in vivo*.

## Results

**Figure 1:**
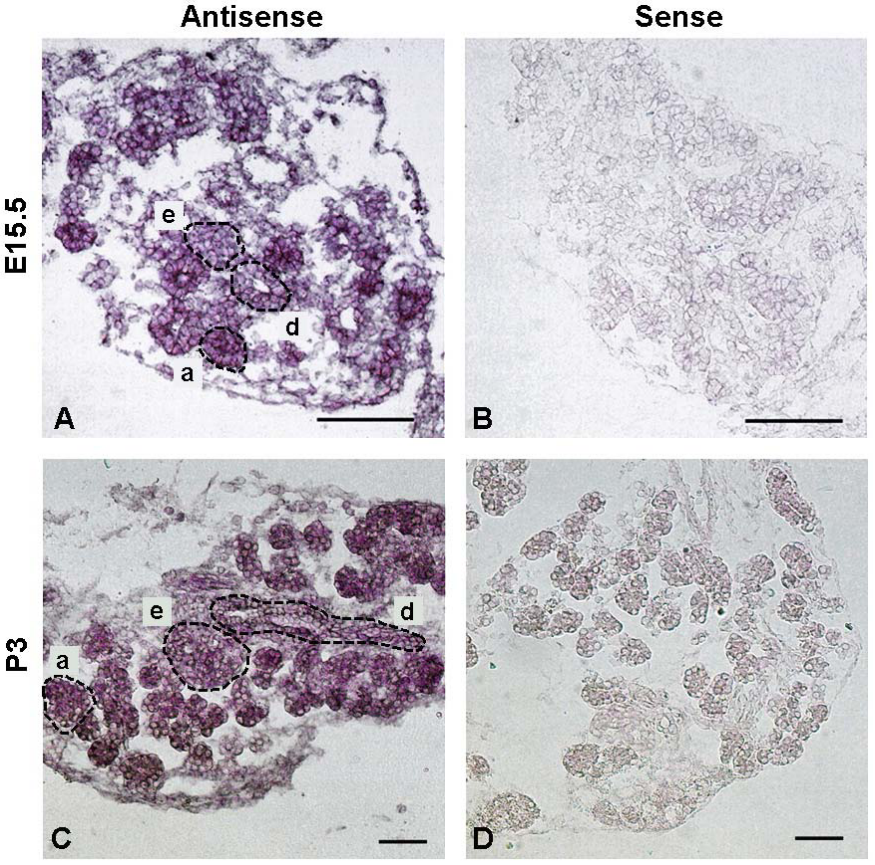
**Expression of Rac1 mRNA in embryonic and neonatal pancreas**. Rac1 mRNA expression was analysed by *in situ *hybridization. Rac1 mRNA is expressed in ducts (d), acinar (a) and endocrine (e) cells in the pancreas both in the developing embryo (E15.5) (A) and in the neonatal pancreas (P3) (C). The levels of Rac1 are highest in the acinar cells. (B,D) Sense probes showing background staining. Bars, 100 μm.

Previously, it was demonstrated that Rac1 mRNA is expressed in the pancreas [17]. Here we show that in the developing pancreas Rac1 mRNA is ubiquitously expressed in all major pancreatic cell types, including ductal, acinar, endocrine and mesenchymal cells at E15.5 and postnatal day (P) 3 (Fig. 1). Notably, the most terminal parts of the ductal tree, including acinar cells, express the highest level of Rac1 mRNA (Fig. 1A,C). Results from microarray analysis of E15.5 embryonic pancreas suggest that the closely related Rac2 and Rac3 are expressed to a much lower extent (<5% of Rac1 expression) (data not shown). We speculate that these low levels of Rac2 and Rac3 mRNAs most likely represent either very low level expression within the epithelium or normal expression within haematopoetic cells [25].

**Figure 2:**
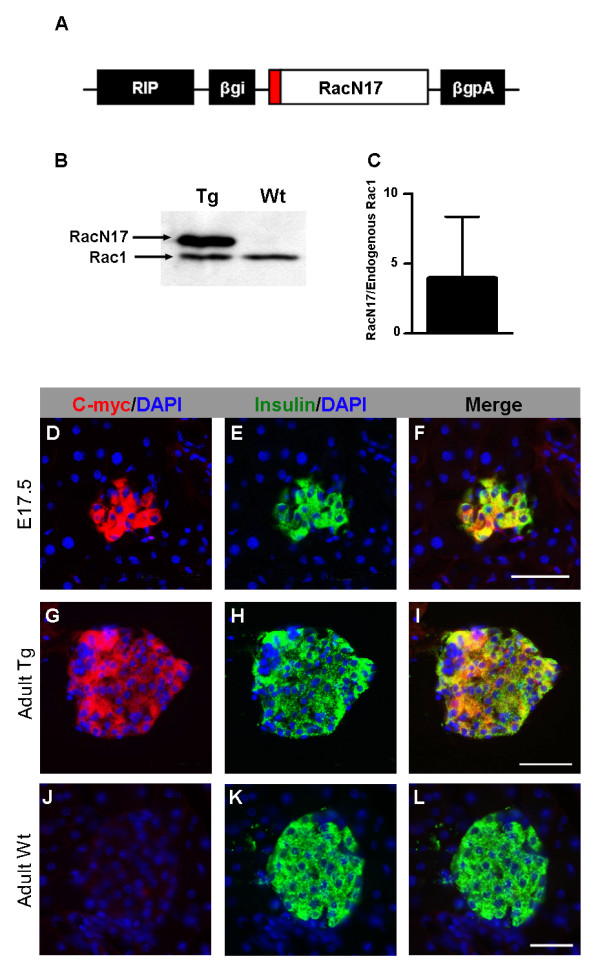
**Construct design and expression of the RIP-RacN17 transgene**. (A) Dominant negative Rac1 (RacN17) was inserted in a vector containing the Rat Insulin Promoter (RIP). A C-myc-tag (red) fused to the N-terminal end of RacN17 enabled monitoring of expression of the transgene. Rabbit β globin intron (βgi) and poly A (βgpA) was inserted to enhance the expression of the transgene. (B) An immunoblot on islets from adult mice demonstrating expression of endogenous Rac1 in wild-type (Wt) islets and expression of endogenous Rac1 and slightly larger RacN17 in transgenic (Tg) islets. (C) Western blot quantification of protein shows that RacN17 is expressed at approximately four fold higher levels than endogenous Rac1 in isolated adult islets (n = 5). (D-L) Immunofluorescence staining on frozen sections with antibodies against C-myc (red) and insulin (green) at E17.5 (D-F) and in the adult (G-I). The transgene is selectively expressed in β cells, and the expression levels tend to vary within the β cell population, especially in the adult. (J-L) Adult wild-type islets show no labelling with C-myc. Error bars represent standard deviation from the mean (± s.d.). Bars, 50 μm.

**Figure 3:**
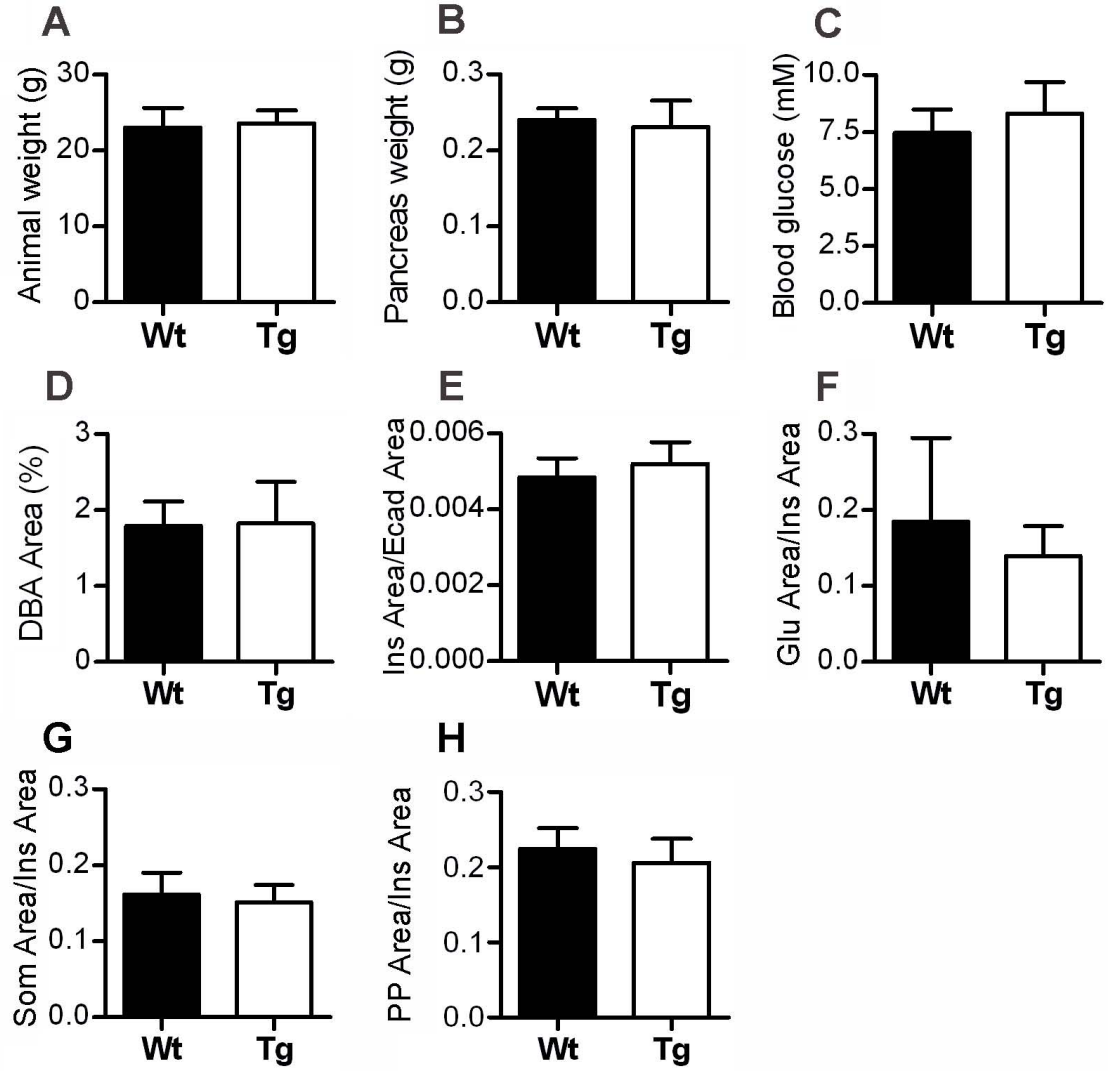
**Body weight, pancreas weight, fasting blood glucose, ductal area, β cell area and islet cell ratios are unaffected in RIP-RacN17 mice**. (A,B) Quantification of body and pancreas weight of six months old female mice showed no difference between transgenic and wild-type littermates (n = 3). (C) Fasting blood glucose levels in 7–9 month old mice (7 Wt male + 11 Tg male) showed no differences between transgenic and wild-type littermates. (D) Measurements of the percentage of pancreas area occupied by DBA-labelled ducts on frozen sections of eight to nine weeks old mice showed no difference between transgenic and wild-type littermates (n = 4). (E) Quantification of β cell area (Ins Area/Ecad Area) in eight to nine weeks old mice showed no difference between transgenic and wild-type littermates (n = 3). (F-H) Measurements of endocrine cell area ratios in eight to nine weeks old mice showed no difference between insulin and glucagon (Glu Area/Ins Area) (F), insulin and somatostain (Som Area/Ins Area) (G) or insulin and pancreatic polypeptide (PP Area/Ins Area) (H) (n = 3). Error bars represent standard deviation from the mean (± s.d.).

To investigate the role of Rac1 in islet morphogenesis, a transgenic mouse model was generated where a C-myc-tagged dominant-negative form of the Rac1 protein was expressed under regulation of the rat insulin promoter [26] (Fig. 2A). Three independent transgenic lines, confirmed by Southern blot analysis (data not shown), expressing comparable levels of the transgene were identified. RacN17 was found to be expressed on average at approximately four fold higher levels compared to endogenous Rac1 (Fig. 2B–C, and data not shown). Because all three lines showed the same phenotype, the presented data is from one line. Control experiments demonstrated that expression of the transgene was restricted to insulin-producing cells both in embryos (E17.5) and adults, and that the expression levels of the transgene varied between individual cells within the islets (Fig. 2D–L). We observed no difference in body weight, pancreas weight or fasting blood glucose levels between wild-type and transgenic littermates (Fig. 3A–C).

### Blocking Rac1 function results in impaired migration of islet cells

**Figure 4:**
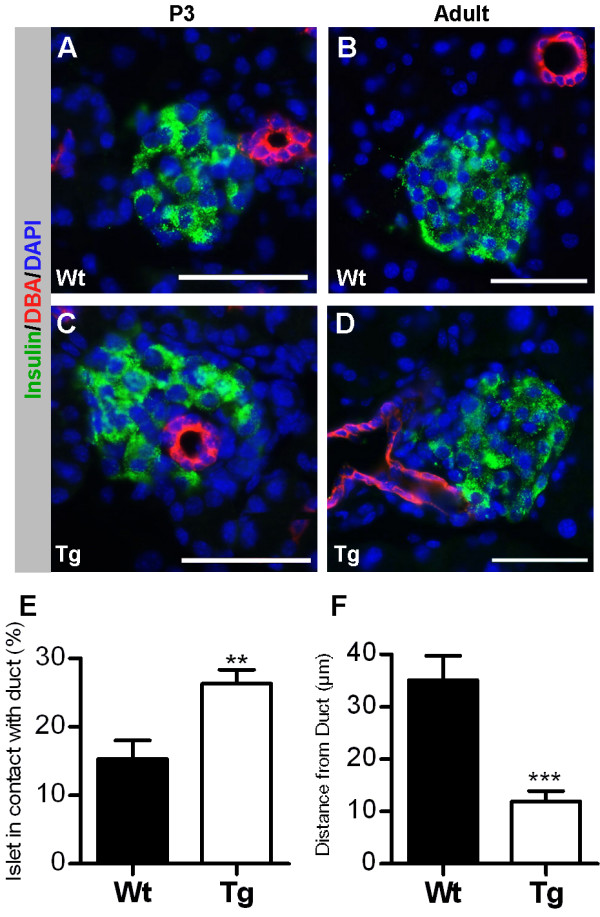
**Blocking Rac1 activation results in impaired migration of β cells**. Frozen sections of P3 (A,C) and adult (B,D) pancreata were stained with antibodies against the ductal marker DBA (red) and insulin (green). The endocrine clusters in the RIP-RacN17 mice (C,D) are found more closely associated with the ducts both at P3 and in the adult compared to wild-type (A,B). (E) Quantification of the percentage of islet circumference in contact with a duct at P3 shows an increase in transgenic mice compared to wild-type littermates, marking a closer association between islets and ducts in transgenic animals (n = 4). (F) Quantification of the distance between the islet and its closest duct in the adult pancreata shows a reduced distance in the transgenic animals compared to wild-type littermates (n = 4). Error bars represent standard deviation from the mean (± s.d.) and significant values are indicated as **p < 0.01 and ***p < 0.0001 determined by Student's t-test. Bars, 50 μm.

Numerous studies have demonstrated an active role of Rac1 in cell migration (reviewed in [16]). Careful analysis of pancreas development in RIP-RacN17 mice revealed no defects preceding islet morphogenesis, the ductal epithelium showed normal branching and differentiation (data not shown). Whereas delamination of newly differentiated β cells and subsequent clustering into vascularised islet-like structures proceeded normally, movement of islets away from ducts was perturbed. The latter was evident from the observation that islets were consistently found more closely associated with ducts compared to wild-type littermates (Fig. 4). Since almost all islets are to some extent in contact with a duct in the neonatal pancreas this phenotype was quantified by estimating the percentage of the islet circumference in contact with a duct at P3, demonstrating a significant increase in islet-duct contact in transgenic mice compared to wild-type littermates (Fig. 4A,C,E). Further analysis showed that this phenotype persisted in the adult (Fig. 4B,D,F). In support of this finding was the observation that mild collagenase-perfusion resulted in a significantly higher number of transgenic islets attached to ducts compared to wild-type islets – a phenotype that could be attributed to stronger islet-ECM interactions (Additional file 1). Importantly, control experiments showed that this phenotype was not secondary to an increased ductal area, i.e. the duct/pancreas ratio was the same in wild-type and RIP-RacN17 mice (Fig. 3D). Furthermore, no changes in β cell area, or endocrine cell ratios were observed (Fig. 3E–H). Finally, careful examination of the final islet morphology and shape revealed more irregularly shaped large islets in RIP-RacN17 mice compared to wild-type littermates (Additional file 2), whereas small and medium sized islets exhibited a normal shape. Altogether, these findings suggest a functional role of Rac1 in islet cell migration and morphogenesis.

### Islet cell spreading *in vitro *depends on Rac1 function

**Figure 5:**
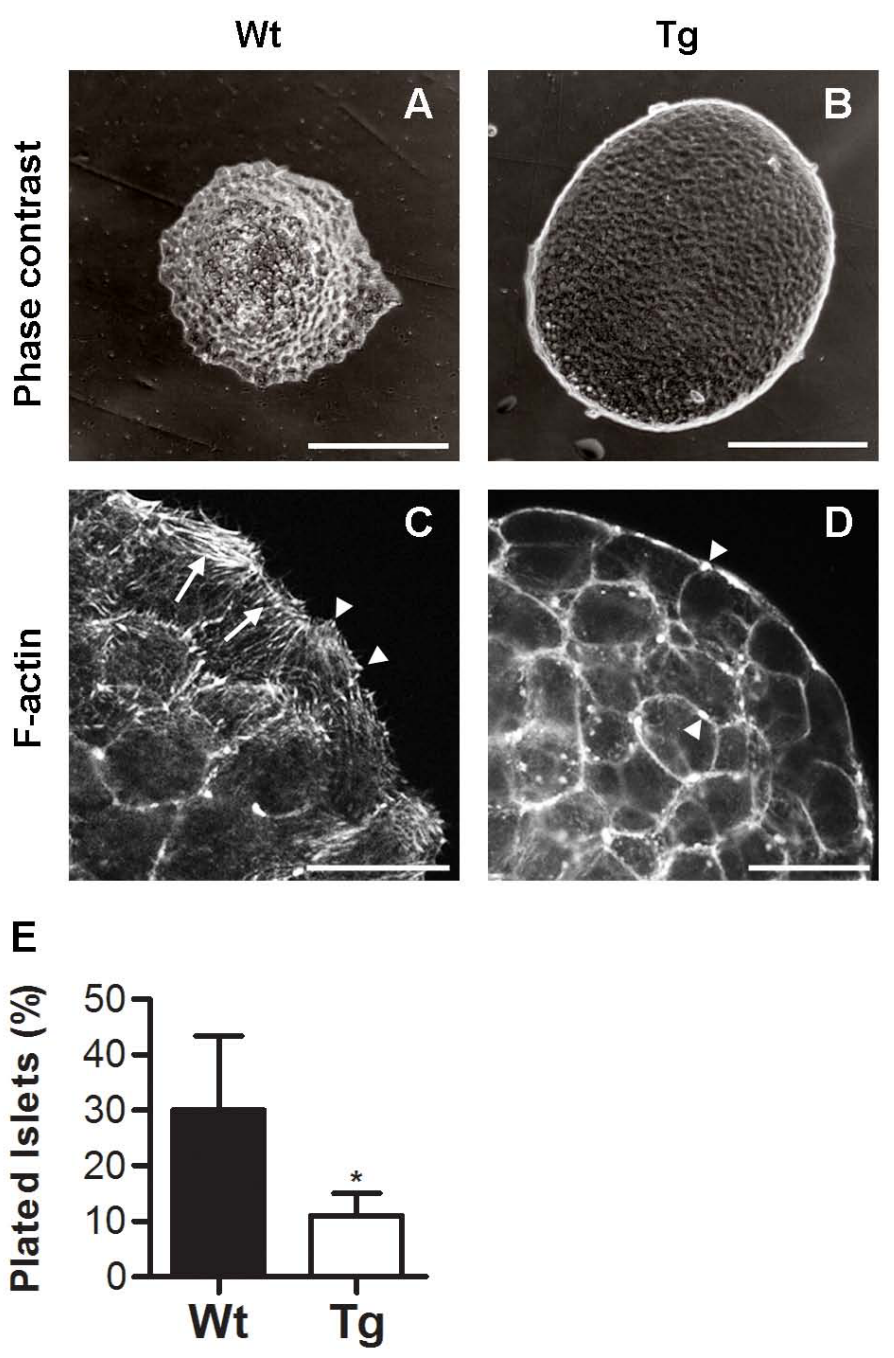
**Blocking Rac1 activation inhibits plating of islets *in vitro***. (A,B) Isolated islets were seeded onto gelatin coated tissue culture dishes and incubated for one week. Whereas wild-type islets plate out and show actin remodelling; marked by phalloidin staining of stress fibres (arrows) of leading-edge cells (C), the RIP-RacN17 islets remained intact (B) with minor actin remodelling (D). (E) Quantification of the plating-efficiency of islets after one week in culture showed lower plating-efficiency of transgenic islets compared to wild-type islets (n = 5). Arrowheads in C and D indicate focal contacts and junctional F-actin, respectively. Error bars represent standard deviation from the mean (± s.d.) and significant values are indicated as *p < 0.05 determined by Student's t-test. Bars, 100 μm (A,B) and 20 μm (C,D).

To further study the role of Rac1 in islet cell migration, attempts were made to develop an *in vitro *islet cell migration assay. For this purpose, islets were dissociated into single cells and seeded onto gelatin-coated plates. However, neither wild-type nor transgenic cells showed any characteristic signs of migratory cells, such as lamellipodia and active remodelling of the actin cytoskeleton, rendering this method unfeasible for comparative studies of cell migration *in vitro *(data not shown). To develop an alternative strategy to study islet cell migration *in vitro*, we considered the fact that *in vivo *movement of islets involve migration of intact islet clusters rather than movement of individual islet cells. Therefore, the *in vitro *behaviour of isolated wild-type and RIP-RacN17 islets plated on gelatin-coated dishes was compared. Normally, islets attach to the surface and after a few days in culture they start to plate out on the surface, changing from a three-dimensional to a two-dimensional structure. Interestingly, although transgenic islets attach to the surface, they plate out less efficiently compared to wild-type islets (Fig. 5A,B,E). Nonetheless, the transgenic islets that do plate out show the same type of actin remodelling as seen in the wild-type (Fig. 5C,D), suggesting that the actin cytoskeletal rearrangements normally involved in the basic migratory machinery is not blocked in all islets.

### Failure to activate Rac1 results in an accumulation of E-cadherin in cell-cell contacts of β cells

**Figure 6:**
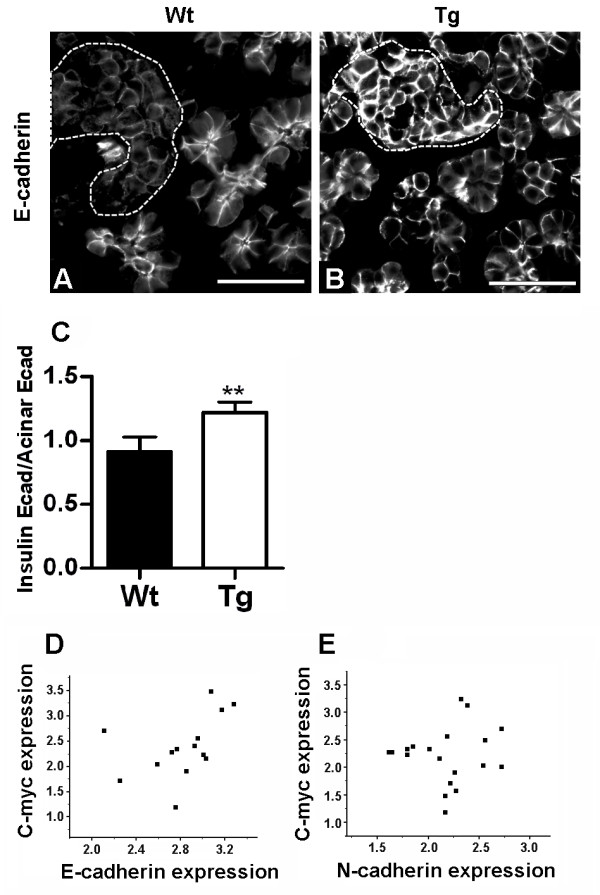
**Deficient activation of Rac1 in β cells correlates with characteristic signs of enhanced cell-cell adhesion**. (A,B) Immunofluorescence staining on frozen sections of P3 pancreata with antibodies against E-cadherin. E-cadherin levels are higher in transgenic (B) compared to wild-type (A) islets. Islets are indicated by dotted lines. (C) Quantification of E-cadherin average staining intensity in P3 mice shows an increase in islet E-cadherin intensity relative to the levels in surrounding acinar tissue in transgenic compared to wild-type pancreata (n = 4). (D,E) Quantitative reverse transcription real-time PCR on single isolated adult β cells shows that E-cadherin but not N-cadherin mRNA correlates with RacN17 mRNA (Values presented as log2 ratios). Ecad-Rac1 shows a Pearson coefficient of 0.70 (if outlier is excluded) (D), whereas Ncad-Rac1 shows a Pearson coefficient of 0.44 (E). Error bars represent standard deviation from the mean (± s.d.) and significant values are indicated as **p < 0.01 determined by Student's t-test. Bars, 50 μm.

Cell-cell adhesion is necessary for movement of cells during tissue formation and organogenesis. To regulate such events the on and off state of cell-cell adhesion must be dynamically regulated. Although cadherin-mediated cell-cell adhesion is crucial for the initial clustering of islet cells [27], its contribution to movement of islets during islet morphogenesis remains unknown. The fact that numerous reports support a regulatory role of Rac1 in E-cadherin-mediated cell-cell adhesion (reviewed in [15]), led us to investigate whether expression of RacN17 affected E-cadherin function. Since isolation of neonatal islets is not feasible, Western blot analysis cannot be used to quantify E-cadherin protein abundance. As an alternative method quantification of E-cadherin staining intensity was used. Indeed, E-cadherin protein was upregulated in transgenic islets at P3 (Fig. 6A–C), whereas β-catenin levels appeared unaffected (data not shown). Quantitative reverse transcription real-time PCR on single isolated adult β cells showed a correlation between E-cadherin and RacN17 expression, i.e. cells expressing higher levels of RacN17 expressed higher levels of E-cadherin (Fig. 6D), suggesting that Rac1 inhibition stimulates *E-cadherin *transcription and/or E-cadherin mRNA stability. Importantly, Rac1 does not seem to exhibit a general effect on cadherin-mediated cell-cell adhesion since no/weak correlation was observed between N-cadherin and transgene mRNAs (Fig. 6E). R-cadherin is also expressed in islets [28], but since it is expressed at low levels and distributed mainly intracellularly (Dahl and Semb, unpublished) it is not considered to contribute to islet cell adhesion.

### Expression and localisation of ECM components remain unaltered in RIP-RacN17 mice

Not only does cell migration require appropriate dynamics of cell-cell interactions, but also precise regulation of attachment and de-attachment to the surrounding ECM. The latter may also require proteolytic cleavage of the basal lamina ECM to get access to the surrounding ECM that function as a substratum for cell migration. To investigate whether the impaired migration of the islets away from the pancreatic ducts in RIP-RacN17 mice could be explained by an excessive deposition of ECM around the islets which would physically prevent movement of the islets, the distribution of various islet ECM components were examined. Consistent with the demonstration that islet ECM is mainly produced by blood vessels, which are recruited to islets during islet morphogenesis [29], no change in the deposition of laminin, collagen IV, and fibronectin were observed (Additional file 3A–F). Alternatively, deficient cell migration could be explained by compromised expression/activity of ECM-receptors such as integrins. However, no change in expression pattern or activity of the major islet integrin-receptor integrin β1 or the adaptor, vinculin, was observed (Additional file 3G–L). In conclusion, these results demonstrate that Rac1 affects islet cell-cell adhesion but appears not to affect expression/distribution of the analysed ECM components.

### Rac1 regulates betacellulin-induced spreading of islets *in vitro*

**Figure 7:**
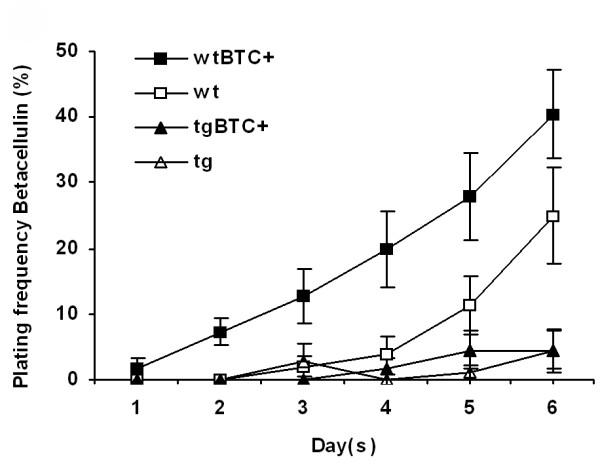
**Betacellulin-stimulated plating of islets is blocked when Rac1 function is inhibited**. Isolated islets were grown in 5% FCS for two days before switched to 0.5% FCS with and without betacellulin (25 ng/ml) (Day 1). Whereas betacellulin stimulated plating of wild-type islets (on average four days to surpass 15% plating-efficiency compared to six days for unstimulated islets, p < 0.05 in a Student's t-test (n = 6)), plating of RIP-RacN17 islets were unaffected (n = 5). Error bars represent standard error of the mean (s.e.m).

To identify the signalling pathway that regulates islet migration through Rac1, two previous observations were of particular interest. First, deficient migration of islets away from ducts was observed in EGF-receptor-deficient mice [10]. Secondly, Rac1 activity has previously been shown to be dependent on EGF-receptor stimulation in other cell types [30]. To investigate whether Rac1 is a downstream signalling component of the EGF-receptor during islet cell migration, isolated islets were cultured in the presence or absence of the EGF-receptor ligand betacellulin. Interestingly, betacellulin stimulated plating of wild-type islets (Fig. 7). Consistent with Rac1 acting downstream of the betacellulin/EGF receptor complex, betacellulin's stimulatory activity on islet plating was blocked in RIP-RacN17 islets. These results suggest that Rac1 acts downstream of the EGF-receptor during betacellulin-induced islet cell spreading.

## Discussion

Although much is known about the cellular and molecular processes that are controlled by members of the Rho-GTPases, there is still a gap in the knowledge of their extracellular effectors and their *in vivo *function during tissue and organ formation, in particular in mammalian organisms. In the developing pancreas, islet morphogenesis, including endocrine cell delamination from ducts and islet cell migration, has not been resolved at a cellular and molecular resolution. The fact that these events most likely involve cellular processes that have been reported to be coordinated by Rac1, such as cell migration and cell adhesion [31], led us to study the functional role of Rac1 in islet morphogenesis. For this purpose, a dominant negative form of Rac1 was expressed in β cells. We show that blocking Rac1 activity in β cells results in compromised migration of islets away from ducts. In fact, more islets remain in direct contact with ducts. The result can neither be explained by a general developmental delay, nor by an increase in the area of the ductal network. Furthermore, we provide evidence that the function of Rac1 in islet cell migration may be controlled by modulation of E-cadherin-mediated cell-cell adhesion. Compromised islet cell migration does not result in a major impact on islet cell function, since fasting glucose levels remained unaltered.

The conclusion that Rac1 is involved in islet cell migration is based on both *in vivo *and *in vitro *observations. *In vivo*, islet cells normally migrate away from ducts subsequent to delamination and clustering. Blocking Rac1 function resulted in deficient migration away from ducts. To study the migration in more detail we used an *in vitro *model where isolated islets were seeded onto gelatin-coated culture dishes. Normally, islets attach and eventually flatten out due to induced cell spreading. Cell spreading is associated with increased formation of cell-ECM interactions (focal contacts) and actin remodelling by leading edge cells. However, blocking Rac1 function in β cells resulted in reduced islet cell spreading. Altogether, the *in vivo *and *in vitro *results suggest that Rac1 is directly involved in the migration of islets and that the underlying mechanism may involve regulation of cell-ECM interactions, cell-cell adhesion, and rearrangement of the actin cytoskeleton. The *in vitro *studies show that betacellulin stimulated islet cell spreading and actin remodelling is compromised in transgenic islets, suggesting that betacellulin may act as a regulator of Rac1 activity and islet migration *in vivo*.

Rac1 has previously been shown to regulate E-cadherin-mediated cell-cell adhesion. However, the picture is rather complex since Rac1 has been reported to both promote and inhibit E-cadherin function. For example, E-cadherin activity is regulated by endocytosis, and Rac1 has been demonstrated to both promote and block E-cadherin endocytosis [22,23]. To explain such contradictory results, it has been speculated that the effect of Rac1 on E-cadherin endocytosis may depend on different downstream effectors due to the cellular context [31]. Here, we provide evidence in support of Rac1 controlling E-cadherin function during pancreatic islet morphogenesis by demonstrating that blocking Rac1 function in β cells results in increased levels of E-cadherin mRNA and protein. Increased levels of E-cadherin at cell-cell contacts may create a more static cell-cell adhesion, which may inhibit the cell movement/rearrangement needed for islets cells to spread *in vitro *as well as for their *in vivo *migration away from ducts. Consistently, recent data demonstrate that the consequence of blocking Rac1 function (either through loss of function mutations or by a dominant negative approach) during Drosophila tubulogenesis, results in perturbed cell rearrangement due to increased E-cadherin protein expression [19,20]. The observed increase of cell-cell contact E-cadherin may also explain the irregular shape of the largest islets in RIP-RacN17 mice. Normally, islets form by fusion of the initial endocrine cell clusters that appear soon after delamination [32]. In order to form the final shape of the mature islet, dynamic cellular rearrangements between neighbouring cells are necessary. Such processes most likely require dynamic changes in cell-cell adhesion and remodelling of the actin cytoskeleton. The existence of large islets with an irregular shape in transgenic mice could either be explained by a failure of smaller islets to separate from each other or by perturbed cellular rearrangement in the largest islets. Altogether, our results support the concept that islet migration away from ducts depend on Rac1-mediated regulation of E-cadherin cell-cell adhesion and actin cytoskeletal remodelling.

It has been suggested that migration of endocrine cells away from the ductal epithelium requires degradation of the basal lamina by MMPs [7]. However, gene ablation studies of MMPs in the developing pancreas showed no effect on islet formation [8]. These results indicate that degradation of the ECM is not necessary for islet migration, suggesting that Rac1's role in islet migration does not involve proteolytic cleavage of ECM components. Furthermore, this conclusion is consistent with the observation that the deposition of basal lamina components, such as laminin and collagen IV, as well as fibronectin was unaffected upon Rac1 inhibition.

To identify in which signalling pathway Rac1 acts as an effector for islet cell migration, we considered the previous findings that EGF-receptor gene ablation results in a similar phenotype with increased association of islets with ducts [10] and that EGF-receptor signalling regulates Rac1 activity [30,33-35]. To establish a potential link between Rac1 and EGF-receptor signalling during islet migration, we tested the activity of the EGF-receptor ligand betacellulin on wild-type and RIP-RacN17 islet cell spreading *in vitro*. These experiments showed that betacellulin stimulates islet cell spreading. The fact that betacellulin was unable to promote spreading of transgenic islets suggests that Rac1 acts as a downstream component in the betacellulin/EGF-receptor signalling pathway during islet cell spreading. Apart from the EGF-receptor (erbB-1) betacellulin also binds to erbB-4. However the fact that the expression of erbB-4 in neonatal and adult pancreas is restricted to ductal- and α-cells [36], suggests that betacellulin signals through erbB-1. Based on these results, we propose a model for islet cell migration where betacellulin/EGF-receptor signalling controls the activity of Rac1, which coordinates changes in E-cadherin-mediated cell-cell adhesion and actin rearrangements to facilitate islet cell movement. This model predicts that the reported deficient migration of islets in EGF-receptor-deficient mice is at least in part due to compromised Rac1 activation.

RacN17 may affect other GTPases [37], thus expression of RacN17 could in theory inhibit other Rac members, such as Rac2 and Rac3. However, Rac2 and Rac3 are not expressed in the pancreas [25,38]. Moreover, RacN17 could affect other Rho-GTPases, such as Cdc42. However, analysis of pancreas specific ablation of Cdc42 shows that the RIP-RacN17 phenotype cannot be attributed to blocking Cdc42 activity (Kesavan and Semb; unpublished). Thus, although we cannot rule out that RacN17 may affect other GTPases in β cells, we believe that the phenotype observed in RIP-RacN17 mice primarily involves Rac1.

Our model predicts a potential link between EGF-receptor signalling and E-cadherin function via Rac1 in β cells. How does Rac1 control the levels of E-cadherin protein in pancreatic β cell-cell contacts? To this end we provide evidence in support of Rac1 controlling E-cadherin transcription and/or mRNA stability. Post-translational effects may also be operational because a recent study showed that EGF induces macropinocytosis of E-cadherin through Rac1 activation [33]. Based on such findings, we speculate that inhibiting Rac1 could block EGF-induced recycling of E-cadherin, resulting in an accumulation of E-cadherin at the cell surface, thereby creating a more static "on-state" of cell-cell adhesion. Consequently, the mechanism by which betacellulin induces migration of islets through Rac1 could tentatively be explained by an effect on E-cadherin recycling – a question to be resolved by future studies.

## Conclusion

In summary, our results provide new insight into how Rac1 controls organogenesis. Specifically, our data support a model where Rac1 signalling controls islet cell migration by modulating E-cadherin-mediated cell-cell adhesion. Furthermore, *in vitro *experiments show that betacellulin stimulates cell spreading and actin remodelling in wild-type but not transgenic islets, suggesting that betacellulin may act as a regulator of Rac1 activity and islet migration *in vivo*. Our results further emphasize Rac1 as a key regulator of cell migration and cell adhesion during tissue and organ morphogenesis.

## Methods

### Generation of transgenic mice

A cDNA with a C-myc-tagged RacN17 [39] was inserted in to a vector containing 0.7 kb of the Rat Insulin Promoter [26] and the rabbit β globin intron and polyA sequences. The construct was cut out by enzymatic digestion and purified before pronuclear injection. Transgenic mice were obtained by pronuclear injection at the Karolinska Center for Transgene Technologies. Founders were confirmed with PCR and Southern Blot. Mice were backcrossed against C57Bl/6J background and wild-type littermates were used as controls. Analysis of three individual founder strains showed that all three strains exhibited the same phenotype. Genotyping was performed by standard PCR-methods using the primers MycMut2, 5'-AGAAGCTGATCTCCGAGGAG-3', and hRac-Rev1, 5'-GAATAATTGTCAAAGACAGTAG-3'. The expression of the transgene was also confirmed by immunohistochemsitry. Animals were maintained in an approved animal facility and all animal work was carried out in accordance with approval by the local ethics committee for animal research.

### Weight and fasting blood glucose level measurements

Female transgenic RIP-RacN17 mice and wild-type littermate controls, six months of age were weighed and pancreas was carefully dissected out before weight measurement. For fasting blood glucose level measurements, male transgenic RIP-RacN17 mice and wild-type littermate controls of seven to nine months were fasted overnight. Blood was obtained from the tail vein and glucose levels were measured using a glucometer.

### *In situ *hybridization

A probe against the 3' end of Rac1 was used (a kind gift from Dr Ivan de Curtis) and was performed as previously described [38]. Briefly, P3 and E15.5 pancreata were fixed in PBS-4% PFA, embedded in Tissue-Tek^® ^O.C.T.™ (Sakura) and 10 μm sections were mounted onto Superfrost^© ^Plus slides (Menzel). Digoxigenin labelling of antisense and sense strand was performed with T3 and T7 RNA polymerase-mediated transcription respectively according to the manufacturers instructions (Roche). Overnight hybridization was carried out with 1 μg/ml of the probe in a solution consisting of 50% deionized formamide, 1 × Denhardt's solution, 10% dextran sulfate, 0.5 mg/ml tRNA and 3 × SSC at 60°C.

### Immunohistochemistry and immunoreagents

For immunohistochemistry, tissues were fixed in PBS-4% PFA overnight at 4°C. For cryo sections, tissues were incubated in PBS-30% sucrose overnight at 4°C, embedded in Tissue-Tek^® ^O.C.T.™ (Sakura) and 10 μm thick sections were mounted on poly lysine coated slides. Sections were blocked in PBS-0.1% Triton X-100 containing 5% non fat dry milk for one hour before incubating with primary antibodies overnight in blocking solution. Slides were incubated with secondary antibodies diluted in blocking solution for one hour before washing and mounting. Immunoreagents used were DBA (1:1000; Vector Laboratories), guinea pig anti-insulin (1:800; Dako), rabbit anti somatostatin (1:500; Sigma), rabbit anti glucagon (1:2000; Linco Research), rabbit anti pancreatic polypeptide (1:200; Euro Diagnostica), DAPI (1:1000; Molecular Probes), rat anti-E-cadherin ECCD-2 (1:300; [40]), mouse anti-C-myc 9E10 (1:200; [41]), rabbit anti-laminin (1:500; Sigma), mouse anti-collagen IV (1:200; Hybridoma bank), rabbit anti-fibronectin (1:300; Dako), mouse anti-vinculin (1:100; Sigma), rat anti-integrin β1 (1:500; Chemicon), rat anti-integrin β1 (active form) (1:750; BD Pharmigen), Alexa Fluor^® ^488 phalloidin (1:500; Invitrogen). Stained sections were photographed in an AxioplanII fluorescent microscope and analysed using Axiovision LE software (Zeiss). Secondary antibodies used included Cy3 anti -rabbit, -rat, -mouse and -streptavidin (Jackson Immuno Research), Alexa Fluor^® ^488 anti-guinea pig (Molecular Probes) and TRITC anti-mouse IgG1 (Southern Biotech).

### Immunoblotting

Islets were boiled for five minutes in Sample Buffer (100 mM TRIS, 4% (w/v) SDS, 0.2% (w/v) bromophenol blue, 20% (w/v) glycerol, 200 mM β-mercaptoethanol), centrifuged for five minutes at 5900 G and samples were separated on SDS-PAGE before transferred onto a nitrocellulose filter (Amersham). The filter was blocked for two hours in PBS-0.05% Tween-20 containing 3% BSA and blotted with mouse anti-Rac1 primary antibody (1:1000; Upstate) overnight at 4°C in blocking solution. The blot was incubated with HRP-conjugated anti mouse secondary antibody (Sigma) for one hour and proteins were visualized by chemiluminescence (Amersham). Quantification of transgenic expression was performed by measuring the ratio of the band intensity on blots between RacN17 and endogenous Rac1 using Image Gauge (Fuji).

### Isolation and plating of islets

Islets and islet cells were isolated according to Olofsson et al. [42]. Briefly, pancreas from sex matched transgenic mice and wild-type littermates two to six months old were perfused with Collagenase P (1.8 U/ml) (Roche) in HBSS, and incubated at 37°C for 20 minutes. For the mild Collagenase P treatment we used a lower concentration of the enzyme (0.57 U/ml). The tissue was dispersed by manual shaking and islets were picked manually. To obtain single cells, isolated islets were incubated in Calcium free media (138 mM NaCl, 5.6 mM KCl, 1.2 mM MgCl_2_, 5 mM HEPES, 3 mM Glucose, 1 mM EGTA 0.1% (w/v) BSA, pH 7.4) at 37°C for 12 minutes and islets were dissociated by pipetting. The cells were washed in HBSS before further usage. Isolated islets were plated onto gelatin coated tissue culture dishes and cultured for seven days in RPMI1640 (Invitrogen) supplemented with 10% FCS. The number of plated islets was determined by daily inspection of the cultures.

For the treatment with the EGF-receptor ligand betacellulin (R&D systems), the islets from each animal were divided into two wells to recover in RPMI with 5% FCS for 48 hours, after which islets were incubated in RPMI with 0.5% FCS with or without 25 ng/ml of betacellulin.

### Quantitative single cell analysis

Gene expression analysis in individual β cells using quantitative reverse transcription real-time PCR was performed as previously described [43]. Individual cells were collected with a glass patch-clamp pipette using a hydraulic micromanipulator. Pipettes were emptied in lysis buffer containing 50 mM Tris-HCl (pH 8.0), 140 mM NaCl, 1.5 mM MgCl_2_, and 0.5% Igepal CA-630. Samples were heated to 75°C followed by vortexing. Tubes were then immediately frozen with dry ice until storage in -20°C. SuperScript III (Invitrogen) was used to generate cDNA using oligo dT and random hexamers as primers according to the manufacturer's instructions. Real-time PCR was measured in the LightCycler (Roche Diagnostics) or the ABI PRISM 7900 (Applied Biosystems) using SYBR Green I (Molecular Probes) as detection chemistry. The correct PCR product was confirmed by melting curve analysis and agarose gel electrophoresis (2% w/v). The primers used were MycMut2; hRacRev1; Ecad forward, 5'-CGCTGAGATGGACAGAGAAG-3'; reverse, 5'-TCTGCTTTGGCTTCCCATCC-3'; Ncad forward, 5'-CAGGTTTGGAATGGGTCTGT-3'; reverse, 5'-ATGTTGGGTGAAGGTGTGCT-3'; Ins II forward, 5'-CCCTGCTGGCCCTGCTCTT-3'; reverse, 5'-AGGTCTGAAGGTCACCTGCT-3'. Ins II was used as a positive control for β cells, and cells not expressing Ins II were excluded from the analysis.

### Morphometric analysis

β cell area was determined by measuring the percentage of the E-cadherin positive pancreas area that is insulin positive on frozen sections separated by 300 μm through the entire pancreas. Animals were between eight and nine weeks of age and transgenic and wild-type littermate pairs of the same sex were used. Endocrine cell ratios was determined by quantifying the ratio of the area occupied by glucagon, somatostatin or pancreatic polypeptide compared to the insulin area on ten islet profiles each from the head and tail of pancreata from eight to nine weeks old mice on frozen sections.

Measurement of the contact between insulin positive islets and DBA positive ducts was used to estimate islet/duct association at P3. To measure the contact between islets and ducts the percentage of the circumference of insulin positive islets in contact with a DBA positive duct on sections was estimated. In total, 50 islets per pancreas were analysed on stained sections separated by 200 μm. Duct distance was estimated by measuring the distance between the islet border, marked by insulin expression and the closest duct, determined by DBA staining and the presence of a lumen. Pancreas from eight to nine weeks old mice were used and 60 islets per pancreas were measured. Sections were separated by 200 μm. Duct area was examined by measuring the percentage of the pancreas area occupied by DBA labelled ductal epithelium on 30 20× images per animal. E-cadherin levels in islets were determined by measuring the average staining intensity of the pixels in the islet relative to acinar cells surrounding the islet on images of 10 μm thick frozen sections. Four regions of acinar cells per islet were measured and six islets per animal were analysed (n = 4). Islet shape was determined by calculating the circularity ratio of 110 islets per animal, marked by a continuous insulin stained area [44]. Briefly, the area (A) of the islet is related to its perimeter (p) in the formula 4πA/p2, which gives a value of one for a perfect circle and the value decreases as the perimeter increases relative to the area indicating an irregular shape of the measured object. The values from the largest ten percent, i.e. the 11 largest islets per animal are presented in the results. Apart from duct association at P3 where ImageJ software was used, all analysis was performed using Axiovision software (Zeiss).

## Authors' contributions

TUG and HS designed the study and drafted the manuscript. TUG generated the transgenic cDNA construct and characterized the transgenic lines. AS performed and analysed the quantitative single cell analysis. TUG and GK performed the *in situ *hybridization, western blot and immunohistochemistry analyses and *in vitro *experiments. All authors reviewed and approved the manuscript.

## Supplementary Material

Additional file 1 **RIP-RacN17 islets attach to ducts after mild collagenase P perfusion**. (A) Mild collagenase P perfusion of adult pancreata resulted in an increased presence of islet-duct aggregates in RIP-RacN17. (B) Quantification of islets that were attached to ducts in adult mice after mild collagenase P perfusion was determined and shows a statistical significant increase in the transgenic animals (n = 4). Error bars represent standard deviation from the mean (± s.d.) and significant values are indicated as **p < 0.01 determined by Student's t-test (n = 4). Bar, 500 μm. Click here for file

Additional file 2 **Deficient activation of Rac1 results in alteration of the shape of large islets**. (A,B) Paraffin sections from adult mice stained with H&E. Wild-type islets (A) show a round or oval shape, whereas transgenic islets exhibit a more irregular shape. (C) Quantification of the shape of the 10% largest islets shows a statistical difference between the wild-type and the transgene. The circularity ratio (4πA/p2) was compared between the wild-type and the transgenic islets (n = 3). Error bars represent standard deviation from the mean (± s.d.) and significant values are indicated as *p < 0.05 determined by Student's t-test (n = 3) (A = area, p = perimeter). Bars, 100 μm. Click here for file

Additional file 3 **Extracellular matrix adhesion and composition remain unaltered in RIP-RacN17 islets**. Immunofluorescence staining of adult pancreata with antibodies against laminin (A,B), collagen IV (C,D), fibronectin (E,F), vinculin (G,H), integrin β1 (I,J) and active integrin β1 (K,L). Bars, 50 μm. Click here for file
