# Supplementary figures and images for: Rac1 regulates pancreatic islet morphogenesis
Source: BMC Dev Biol. 2009 Jan 6;9:2. doi: 10.1186/1471-213X-9-2 (PMC2628879; doi:10.1186/1471-213X-9-2)

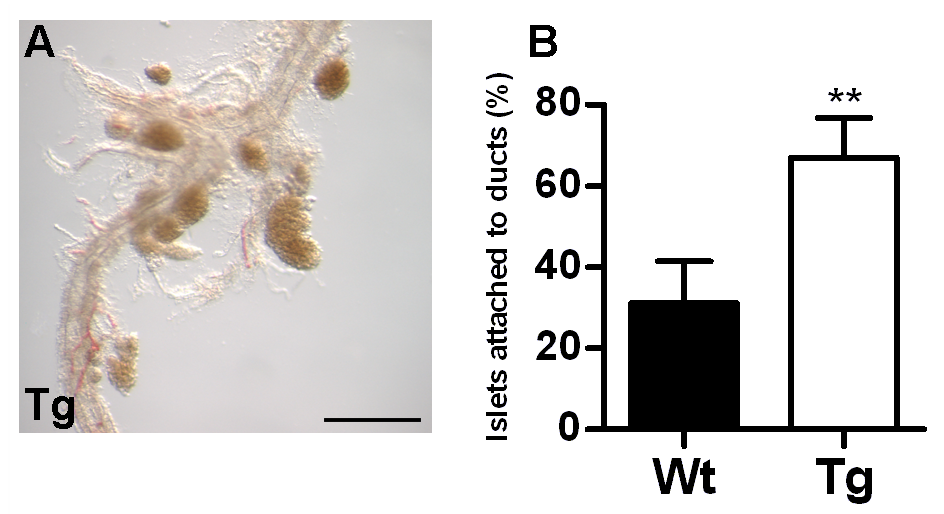

Supplement: Additional file 1 — RIP-RacN17 islets attach to ducts after mild collagenase P perfusion. (A) Mild collagenase P perfusion of adult pancreata resulted in an increased presence of islet-duct aggregates in RIP-RacN17. (B) Quantification of islets that were attached to ducts in adult mice after mild collagenase P perfusion was determined and shows a statistical significant increase in the transgenic animals (n = 4). Error bars represent standard deviation from the mean (± s.d.) and significant values are indicated as **p < 0.01 determined by Student's t-test (n = 4). Bar, 500 μm. [file 1471-213X-9-2-S1.tiff]

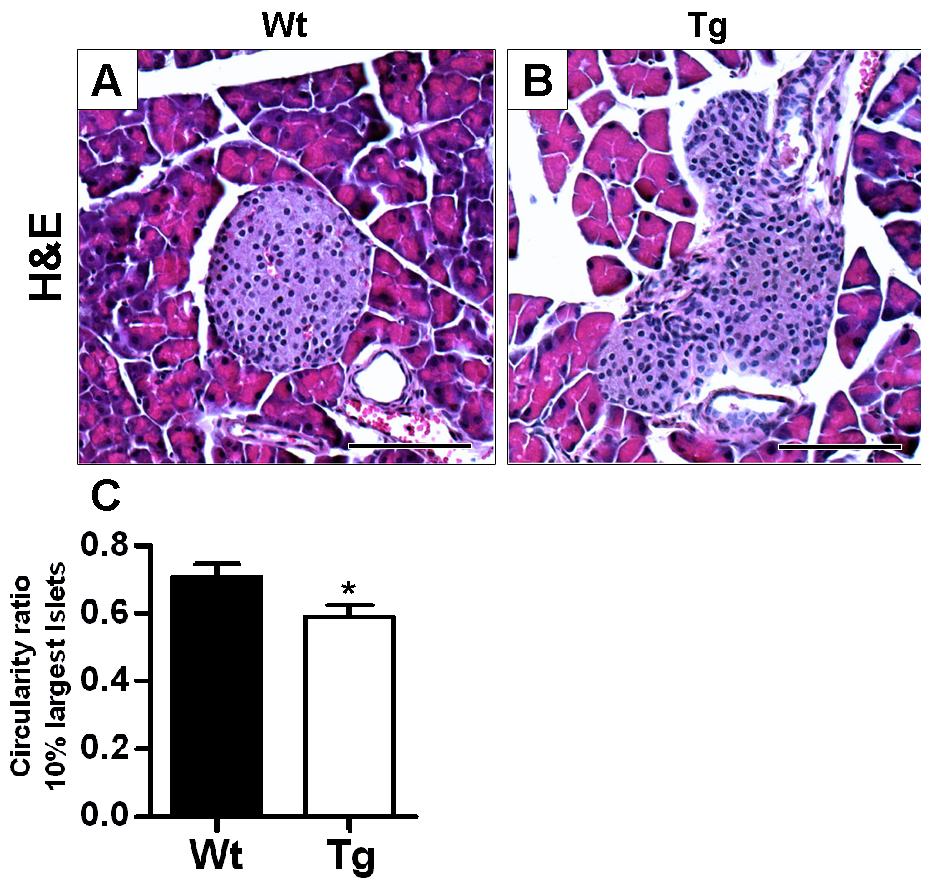

Supplement: Additional file 2 — Deficient activation of Rac1 results in alteration of the shape of large islets. (A,B) Paraffin sections from adult mice stained with H&E. Wild-type islets (A) show a round or oval shape, whereas transgenic islets exhibit a more irregular shape. (C) Quantification of the shape of the 10% largest islets shows a statistical difference between the wild-type and the transgene. The circularity ratio (4πA/p2) was compared between the wild-type and the transgenic islets (n = 3). Error bars represent standard deviation from the mean (± s.d.) and significant values are indicated as *p < 0.05 determined by Student's t-test (n = 3) (A = area, p = perimeter). Bars, 100 μm. [file 1471-213X-9-2-S2.tiff]

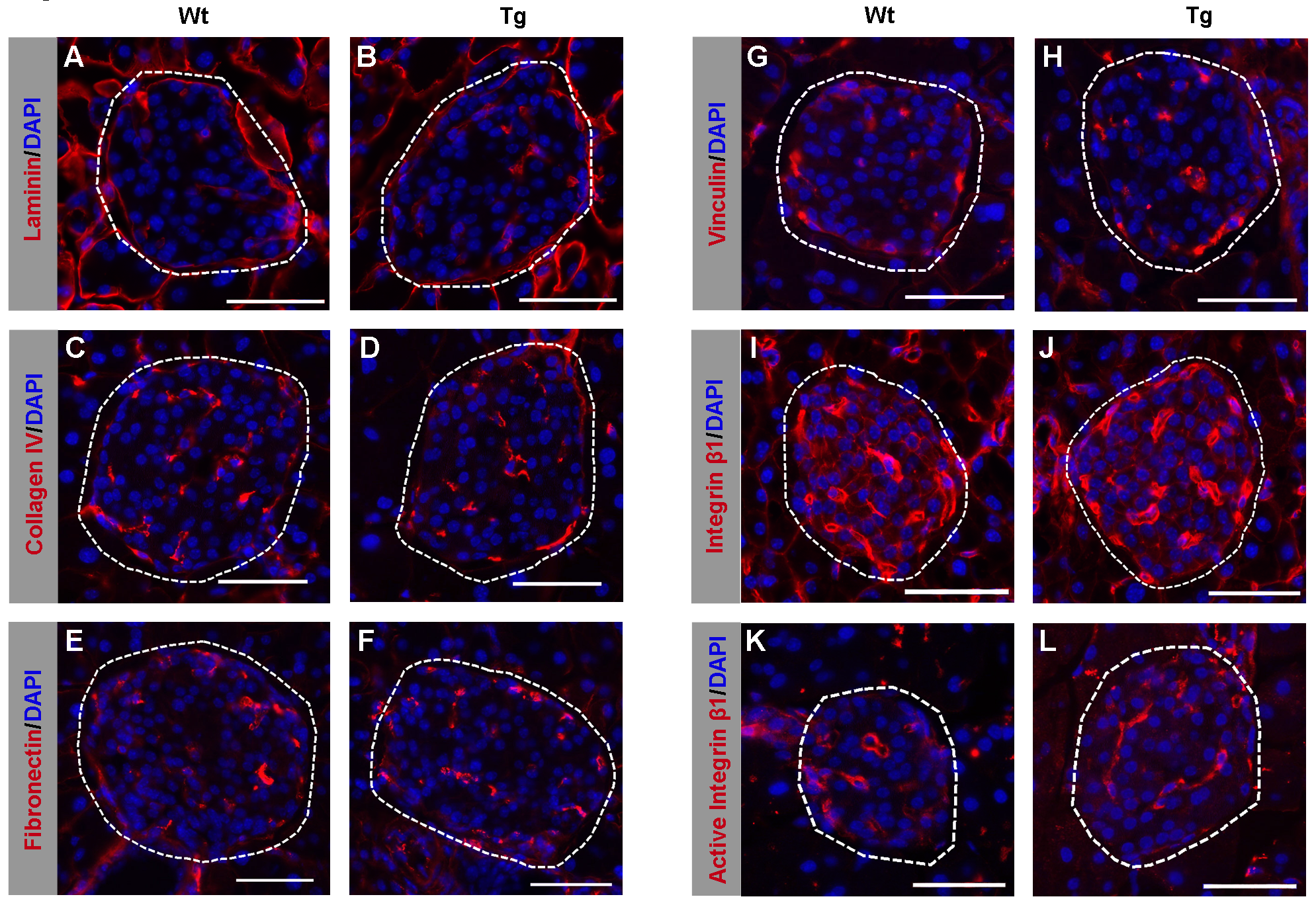

Supplement: Additional file 3 — Extracellular matrix adhesion and composition remain unaltered in RIP-RacN17 islets. Immunofluorescence staining of adult pancreata with antibodies against laminin (A,B), collagen IV (C,D), fibronectin (E,F), vinculin (G,H), integrin β1 (I,J) and active integrin β1 (K,L). Bars, 50 μm. [file 1471-213X-9-2-S3.tiff]
